# Chronic recurrent multifocal osteomyelitis and primary sclerosing cholangitis with type 1 autoimmune hepatitis in a child with ulcerative colitis: a case report

**DOI:** 10.1186/s41927-021-00186-3

**Published:** 2021-06-03

**Authors:** Hon Yan Ng, Orlee R. Guttman, Lori B. Tucker

**Affiliations:** 1 Division of Rheumatology, Department of Pediatrics, University of Saskatchewan, Saskatoon, Canada; 2 Edmonton, Canada; 3 Division of Gastroenterology, Hepatology and Nutrition, Department of Pediatrics, University of British Columbia, Vancouver, Canada; 4 Division of Rheumatology, Department of Pediatrics, University of British Columbia, Vancouver, Canada

**Keywords:** Chronic nonbacterial osteomyelitis, Primary Sclerosing cholangitis, Autoimmune hepatitis, Ulcerative colitis, Case report

## Abstract

**Background:**

Chronic Recurrent Multifocal Osteomyelitis (CRMO) is a condition characterized by sterile bone inflammation, usually occurring in childhood. Although the etiology remains unclear, this condition has been associated with inflammatory bowel disease (IBD). Primary sclerosing cholangitis (PSC) and Autoimmune Hepatitis (AIH) are also uncommon pediatric conditions with a known association with IBD.

**Case presentation:**

We present a unique case of a pediatric patient with an initial diagnosis of CRMO, with subsequent diagnosis of autoimmune hepatitis and PSC overlap, and eventually IBD.

**Conclusions:**

Patients with CRMO may also develop PSC in addition to IBD, further highlighting the importance of IBD pathophysiology in both conditions. Clinical screening of associated gastrointestinal findings may be of value in patients with CRMO.

## Background

Chronic Recurrent Multifocal Osteomyelitis (CRMO), first described by Gideon et al., is a rare childhood condition characterized by sterile inflammation of the bone [1], and has been also referred to as “chronic nonbacterial osteomyelitis”, and “chronic sclerosing osteitis” [2]. This condition usually occurs in childhood, with affected areas anywhere in the skeletal system, although metaphyseal long bones such as distal and proximal tibia, clavicle, and humerus are commonly involved. Patients may present with bone pain, swelling, local tenderness, and constitutional symptoms [3]. Laboratory changes can be seen including leukocytosis, and raised inflammatory markers and immunoglobulins [4]. The etiology is unclear but thought to be an autoinflammatory disorder due to IL-1 dependent phenotype in murine models [5]. CRMO has been described in association with Inflammatory Bowel Disease (IBD), psoriasis, and Synovitis, Acne, Pustulosis, Hyperostosis, Osteitis Syndrome (SAPHO), leading to the postulation that it belongs to the spectrum of spondyloarthropathies and potentially can be an extraarticular manifestation of IBD [3].

Primary sclerosing cholangitis (PSC) is a rare condition in pediatrics with an incidence of 0.2 cases per 100,000 children [6], characterized by chronic, progressive fibrosis of the intrahepatic and extrahepatic bile ducts. Although the etiology is unclear, there is also a high concordance rate with IBD, especially Ulcerative Colitis (UC) [6, 7]. To our knowledge, PSC, UC, and CRMO have never been reported together before. We report a child with an initial diagnosis of CRMO, with subsequent diagnosis of autoimmune hepatitis/PSC overlap, and eventually UC. The association of these 3 diseases may lend insight towards their pathogenesis.

## Case presentation

A previously healthy 12-year-old girl presented with a 2-year history of chronic progressive bilateral knee pain, worsening in the preceding 6 months. Her symptoms were episodic and variable in duration and severity, with difficulty participating in sports. She had a 2.5 kg weight loss over 2 months and low BMI of 14 kg/m^2^. She denied any gastrointestinal symptoms, oral ulcers, skin changes, or ocular symptoms. She had not tried any specific treatments or interventions. She is of South Asian background and the product of a non-consanguineous relationship. There is no relevant family history of CRMO, autoinflammatory disease, or IBD. Examination showed fullness and tenderness in both medial femoral condyles. Her abdominal and perianal examinations were benign.

**Fig. 1:**
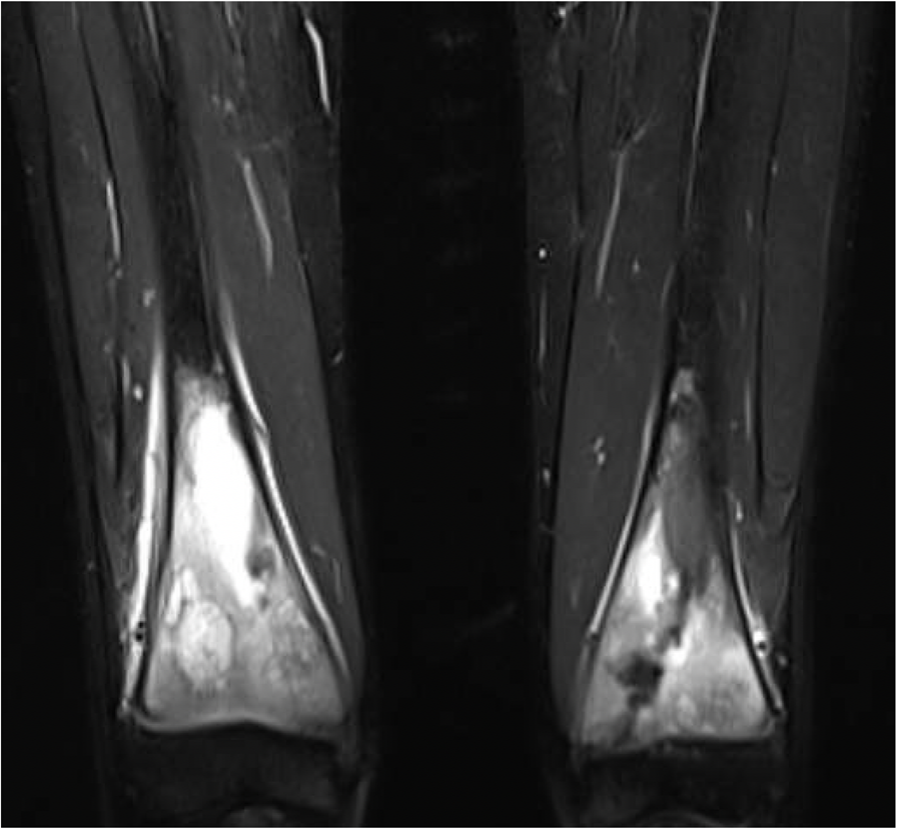
Lower Extremity MRI (STIR) showing multifocal distal femoral abnormalities with regional bone marrow edema pattern, cortical thickening, and periostitis

Initial investigations showed a normocytic anemia (Hb 112 g/L, MCV 79 fL), raised transaminases (ALT 84, AST 81, ALP 227, GGT 146 U/L), and raised inflammatory markers (ESR 94 mm/hr., CRP 15 mg/L). X-rays of the hips, femur, and knees showed distal femoral metaphyseal lytic lesions with surrounding sclerosis, and MRI of the lower limbs revealed multifocal distal femoral bone marrow abnormalities with regional edema pattern, cortical thickening, and periostitis (Fig. 1). A whole-body MRI revealed additional bone marrow edema pattern involving bilateral medial clavicular heads and right acromion.

The patient was treated with a single dose of intravenous zoledronic acid (0.0125 mg/kg) with significant clinical improvement and improved mobilization, but demonstrated persistently abnormal liver enzymes (AST 71, ALT 67, GGT 124 U/L), anemia (Hb 106 g/L), and raised inflammatory markers (ESR 82 mm/hr) and gamma globulins (IgG 30.1 g/L, IgM 3.3 g/L). Conjugated bilirubin was < 0.2umol/L, albumin was 42 g/L, and INR was 1.0. The patient remained asymptomatic without abdominal pain or bowel alterations, jaundice, or pale stools.

Further workup with pediatric gastroenterology was concerning for Type 1 Autoimmune Hepatitis (AIH) with Anti-Smooth muscle > 1:640, ANA negative, Anti-LKM negative, and ANCA negative. Abdominal ultrasound showed mildly heterogeneous liver echotexture but a normal biliary tree; magnetic resonance cholangiopancreatography was normal. Transient elastography showed increased liver stiffness (8.8 kPa, IQR/median of 7%). Liver biopsy showed features of both small-duct PSC and AIH, with interface hepatitis with plasma cells, concentric fibrosis of bile ducts, grade 3–4 hepatitis and stage 3–4 fibrosis. Other diagnoses including hepatitis B/C and tuberculosis, Wilson disease and celiac disease were excluded.

Although the patient was asymptomatic, given the strong association between PSC and IBD, the patient’s fecal calprotectin was measured. This was elevated at 615 μg/g, and so she underwent gastroscopy and colonoscopy. This showed chronic mildly active colitis (non-granulomatous) from cecum to rectum with normal terminal ileum, and normal upper endoscopy, leading to a diagnosis of ulcerative colitis.

The patient’s liver and gastrointestinal disease was treated with oral prednisone (35 mg oral daily), azathioprine (75 mg oral once daily), and ursodeoxycholic acid (250 mg oral daily) with sufficient adherence. No reported adverse effects were reported. There was normalization of liver biochemistry and liver stiffness (5.5 kPa with IQR/median 19%) within 6 months. The patient’s musculoskeletal symptoms remain inactive 11 months since initial therapy with zoledronic acid.

## Discussion and conclusions

We present a child with an initial presentation of CRMO, who was subsequently diagnosed with autoimmune hepatitis/primary sclerosing cholangitis and ulcerative colitis. To our knowledge, this constellation of diagnoses has never been reported. CRMO and autoimmune hepatitis/sclerosing cholangitis have never been reported together, making the initial diagnosis of two seemingly unrelated conditions unusual. Despite this, both conditions are individually associated with IBD.

CRMO is known to be associated with IBD, with reports of musculoskeletal manifestations typically predating IBD between 3 months to 7 years [7]. Other extraarticular associations of CRMO include fever, psoriasis, palmoplantar pustulosis, and Sweet Syndrome [4]. It is unclear whether patients with CRMO and IBD have a different phenotype than those with CRMO alone. The musculoskeletal manifestations typically respond well to the same treatment as IBD, leading authors to postulate a common pathophysiology [8]. Our patient’s presentation appears consistent with those with concurrent CRMO and IBD as the musculoskeletal manifestations predated IBD diagnosis. The patient’s CRMO became inactive 4 months after treatment with zoledronic acid, although the addition of immunosuppressive therapy for AIH/IBD may also have contributed to disease remission. This finding is consistent with reports by Bousvaros et al., showing increased CRMO activity when IBD is active [8].

The pathogenesis of CRMO and its possible relationship to IBD is unclear. Murine models of CRMO suggest that this condition falls along the spectrum of autoinflammatory disorders, similar to those characterized by bone inflammation. These models show that PSTPIP2 gene deficiency leads to aberrant IL-1 signaling as seen in inflammatory bone diseases including Deficiency of IL-1 Receptor Antagonist and Cherubism, supporting the importance of IL-1 in the CRMO phenotype [5]. CARD15 gene is responsible for encoding NOD2, a ubiquitous pattern recognition receptor whose functions are important for immune regulation. Although the CARD15 variants are implicated in the development of Crohn disease (CD), recent studies in patients with both CD and CRMO did not show an increased rate of CARD15 polymorphism [9]. CRMO has also been thought to belong to the spectrum of spondyloarthropathies, due to the observation of association with IBD, sacroiliitis, psoriasis, and its adult counterpart SAPHO syndrome [2].

Primary sclerosing cholangitis is strongly associated with IBD: 76% of patients have IBD, among whom 83% have the UC subtype [6]. Among children, 33% of PSC patients also have AIH, of which 97% are type 1 disease. In pediatric patients with IBD, the association between PSC and AIH further increases to 63% of PSC cases [6]. Other reported immune-mediated associations include celiac disease, Hashimoto thyroiditis, Juvenile Idiopathic Arthritis, and vitiligo. Despite the high rate of concordance, the pathophysiology is not fully elucidated. Both UC and PSC share high frequencies of p-ANCA autoantibody, up to 68% in UC and 85% in PSC [10]. Other theories include the “leaky gut” hypothesis which postulates that microbiome dysbiosis in IBD leads to translocation of bacterial metabolites to the liver causing progressive hepatobiliary injury [11]. Genome-wide Association Studies (GWAS) showed distinct susceptibility loci in both conditions but surprisingly limited overlap to explain the correlation [12]. Although GWAS shows limited overlap, further genetics evaluation for our patient may lend insight towards the association of these 3 conditions and guide therapy.

Although PSC has never been reported with CRMO, there are rare reported cases of PSC with SAPHO, the adult counterpart to CRMO. A large systematic review examining SAPHO syndrome and IBD association identified a total of 39 patients with SAPHO and IBD overlap. Within this cohort, 2 patients also had PSC [13]. As PSC and its relationship to IBD is mostly described in literature on adult patients [14], perhaps the PSC and CRMO association is being captured in the adult population in the form of SAPHO. To our knowledge, AIH has not been reported with SAPHO.

In summary, our report describes the previously undocumented association of AIH/PSC and CRMO. Interestingly, PSC has been rarely documented in patients with both IBD and SAPHO, the adult counterpart of CRMO. As both PSC and CRMO have also been individually associated with IBD, this highlights the importance of IBD pathophysiology in both conditions. As the diagnosis of CRMO may predate IBD presentation, patients with recently diagnosed CRMO may benefit from monitoring for evidence of IBD and its associated conditions, PSC and AIH. Since adults with SAPHO have been reported to develop PSC, we hypothesize that CRMO patients may potentially develop PSC later in life. Long-term follow-up of these patients will be valuable in determining whether they follow the same natural history as their counterparts without gastrointestinal or liver disease.

## Data Availability

Data sharing is not applicable to this article as no datasets were generated or analyzed.

## Abbreviations

CRMO: Chronic Recurrent Multifocal Osteomyelitis
IBD: Inflammatory Bowel Disease
SAPHO: Synovitis, Acne, Pustulosis, Hyperostosis, Osteitis Syndrome
PSC: Primary Sclerosing Cholangitis
UC: Ulcerative Colitis
AIH: Autoimmune Hepatitis
CD: Crohn’s Disease
GWAS: Genome-wide Association Studies
